# Gene Expression Analysis of Human Papillomavirus-Associated Colorectal Carcinoma

**DOI:** 10.1155/2020/5201587

**Published:** 2020-03-17

**Authors:** Qiancheng Qiu, Yazhen Li, Zhiqiang Fan, Fen Yao, Wenjun Shen, Jiayu Sun, Yumeng Yuan, Jinghong Chen, Leshan Cai, Yanxuan Xie, Kaixi Liu, Xiang Chen, Xiaoyang Jiao

**Affiliations:** ^1^The First Affiliated Hospital of Shantou University Medical College, Shantou, Guangdong 515041, China; ^2^Department of Cell Biology and Genetics, Shantou University Medical College, Shantou, Guangdong 515041, China; ^3^Jiangmen Central Hosptial (Affiliated Jiangmen Hospital of Sun Yat-Sen University), Guangdong 529000, China; ^4^Department of Pharmacology, Shantou University Medical College, Shantou, Guangdong 515041, China; ^5^Center for Disease Control and Prevention of Shantou, Guangdong 515041, China; ^6^Shantou Central Hospital, Shantou, Guangdong 515041, China

## Abstract

**Purpose:**

Human papillomavirus (HPV) antigens had been found in colorectal cancer (CRC) tissue, but little evidence demonstrates the association of HPV with oncogene mutations in CRC. We aim to elucidate the mutated genes that link HPV infection and CRC carcinogenesis.

**Methods:**

Cancerous and adjacent noncancerous tissues were obtained from CRC patients. HPV antigen was measured by using the immunohistochemical (IHC) technique. The differentially expressed genes (DEGs) in HPV-positive and HPV-negative tumor tissues were measured by using TaqMan Array Plates. The target genes were validated with the qPCR method.

**Results:**

15 (31.9%) cases of CRC patients were observed to be HPV positive, in which HPV antigen was expressed in most tumor tissues rather than in adjacent noncancerous tissues. With TaqMan Array Plates analyses, we found that 39 differentially expressed genes (DEGs) were upregulated, while 17 DEGs were downregulated in HPV-positive CRC tissues compared with HPV-negative tissues. Four DEGs (MMP-7, MYC, WNT-5A, and AXIN2) were upregulated in tumor vs. normal tissues, or adenoma vs. normal tissue in TCGA, which was overlapped with our data. In the confirmation test, MMP-7, MYC, WNT-5A, and AXIN2 were upregulated in cancerous tissue compared with adjacent noncancerous tissue. MYC, WNT-5A, and AXIN2 were shown to be upregulated in HPV-positive CRC tissues when compared to HPV-negative tissues.

**Conclusion:**

HPV-encoding genome may integrate into the tumor genomes that involved in multiple signaling pathways. Further genomic and proteomic investigation is necessary for obtaining a more comprehensive knowledge of signaling pathways associated with the CRC carcinogenesis.

## 1. Introduction

Colorectal cancer (CRC) is the primary cause of cancer mortality. Although progress has been made, the long-term survival of patients with metastatic disease is still poor [1]. Both genetic and environmental factors contribute to the pathogenesis of CRC; about 75% of CRC cases are not hereditary and occur spontaneously [2]. The gut microbiota is closely correlated with the progression of CRC [3, 4]. The dysbiosis is associated with the genesis and evolution of CRC, but the relationship remains unclear [5–8]. Accumulating evidence shows that colonizing microbes can drive cancer development and progression by direct or indirect effects on host tissues, potentially through inflammatory pathways or carcinogenic microbial metabolites; a causative link was found between chronic inflammation and CRC [9–11]. As microorganisms establish a persistent infection in host cells, either in the form of silent or active infections, barrier deterioration may be a major contributor to colorectal tumorigenesis by microbial products that trigger tumor-elicited inflammation [12, 13].

HPV carries out its life cycle in either the mucosal or the cutaneous stratified squamous epithelia. An oncogenic HPV infection is associated with several malignancies, including cervical cancer [14, 15] and lung cancer [16]. To date, 200 distinct HPV genotypes have been identified, of which at least 18 belong to the “high-risk” group that is chiefly responsible for the development of cancer [17, 18]. Recently, studies have demonstrated the possible involvement of HPV in CRC [19], owing to the detection of HPV antigen or DNA in CRC tissues [20–22]. However, the findings are not consistent. HPV DNA was positive in 60 (83.3%) of the 72 total cancerous colorectal samples, and no HPV DNA was observed in any of the noncancerous tissues in one study [22], while in another study, HPV DNA also found in 53% of healthy mucosal tissues [23]. The frequency of HPV DNA in tumor tissues was higher than that in nontumor colorectal tissues in a Chinese population [24]. Evidence of HPV existing in CRC suggests its role in CRC carcinogenesis. Persistent with a high-risk type of HPV infection is responsible for the premalignant lesions and progression of CRC, which correlated with the late clinical stage [25–27]. Theoretically, the effect of a virus on cancer may be mediated through the integration of the mutagenic viral genome into the host genome, expression of oncogenic viral proteins, or inhibition of tumor-suppressive genes [28–30]. HPV-encoding genome may integrate into the tumor genome, which is considered an essential step in the malignant progression [31]. Currently, there is little evidence demonstrating the association of HPV infection with oncogenic mutations in CRC [32], and the type of oncogenes involved with HPV remains to be elucidated.

Gene analysis with microarray technology has shown great potential in discriminating sophisticated gene profiling, simultaneously mapping thousands of genes in a single sample, and giving a measurement of articulated gene expression patterns [33]. To clarify the molecular mechanism underlying HPV-associated gene mutations in CRC, we used TaqMan Array Plates to detect differentially expressed genes (DEGs) in HPV viral-positive and HPV viral-negative CRC tissues. To overcome the limitation of the small sample size, we also took advantage of the bioinformatics technique; the significant genes associated with CRC screened among the pool of all DEGs in The Cancer Genome Atlas (TCGA). Finally, we validated target genes in CRC tissues by conventional quantitative assays (RT-PCR) and aimed to elucidate whether there is a specific gene mutation pattern involving HPV-related CRC. Identification of genome aberrations in CRCs may help to clarify the mechanism of pathogenesis involved with virus-related CRC.

## 2. Material and Methods

### 2.1. Ethics Statement

The Ethics Committee approved this project of the Shantou University Medical College Ethics Board. This study was conducted under the Declaration of Helsinki.

### 2.2. Patients

All clinical samples were obtained from patients admitted to the First Affiliated Hospital of Shantou University Medical College. Written informed consent was obtained from all patients. The CRC patients were selected based on histologically confirmed CRC at TNM stage I-IV disease with or without distant metastases. An individual with a history of polyps, adenomas, or other diseases related to cancer was excluded. Tumor types were evaluated according to histology, immunohistochemistry (IHC), and chromatin assays. Clinical and histopathologic staging at diagnosis was determined in all patients by combining histopathologic findings with surgical records and operative imaging. All selected tissues were based on histopathological diagnoses; adjacent noncancerous tissue was collected from an area that was 15 cm distal from the tumor. Fresh frozen tissues were obtained from CRC patients before they underwent curative surgical resection. Tumor and the nontumor regions were distinguished using H&E matched slides and microdissected to pinpoint the tumor as well as nontumor areas. Samples were kept at -80°C until measurement. The demographic, clinical, and histopathological data of patients were recorded. Clinical data including race, gender, associated past medical history, medication use, family history of colonic cancer, and laboratory tests were collected for all patients.

### 2.3. Immunohistochemical Analyses

Immunohistochemical staining was performed on paraffin sections 4 *μ*m thick. The sections were cut, dewaxed in xylene, and rehydrated in decreasing concentrations of ethanol. HPV was measured in the tumor tissues and tumor-adjacent tissues using an IHC technique according to the manufacturer's instructions. Considering the HPV's pathogenesis, we had chosen the anti-HPV high-risk subtype antibodies, which is a monoclonal antibody, reacting with HPV subtypes 6, 11, 16, 18, 31, 33, 42, 51, 52, 56, and 58. The details of antibody are HPV mouse monoclonal antibody IgG1, ab75574, Lot. GR207368-5, Abcam, U.K. In negative control sections, the primary antibody was omitted. Virus-infected monocytes or lymphocytes from the same patient and cervical squamous cell carcinoma were used as the positive control for staining. The sections were graded based on the estimated percentages of virus protein-positive cells. IHC staining was evaluated and graded by two pathologists in isolation from the investigator who performed the real-time PCR.

### 2.4. CRC-Associated Oncogene Measurement

Genes expressed in HPV-positive and HPV-negative tumor tissues were measured by using TaqMan Array Plates. Fresh specimens were obtained from the initial diagnosis of CRC patients who underwent curative surgical resection; adjacent noncancerous tissue was taken far mostly from the tumor. Total RNA was extracted from the tissue using a PureLink RNA Mini Kit (Applied Biosystems, USA) according to the manufacturer's instructions. The RNA concentration was measured using the NanoDrop 1000 spectrophotometer (Thermo Fisher Scientific), and RNA quality was evaluated by using the BioAnalyzer 2100 microcapillary electrophoresis system (Agilent Technologies, Inc., Santa Clara, CA, USA). The RNA concentration was adjusted 75 ng/L, so that the RNA concentration of each sample was consistent, and then, reverse transcription was performed. RNA was converted into cDNA by using a High-Capacity cDNA Reverse Transcription Kit (Applied Biosystems, USA). The cDNA microarray analysis was performed using the TaqMan® Array Human Colorectal Cancer Metastasis 96-well plate that contains 92 assays for CRC metastasis-associated genes and four assays to candidate endogenous control genes. Each plate includes predefined probes and endogenous controls, ready for accurate assessment of an entire gene signature in one simple experiment (Thermo Fisher Scientific). Each patient's sample was measured in the tumor tissues and tumor-adjacent tissues simultaneously, and the gene expression level was calculated by gene level in tumor tissue minus gene level in tumor-adjacent tissue. Each cDNA was analyzed using real-time PCR on the “mixed” TaqMan Array Cards, and Ct values for target genes were obtained. All Ct values were normalized with an inner control (GAPDH) gene, and ΔCt values were calculated. Relative gene expression was computed using the relative expression level for each gene. The 2ΔCt values were calculated by SDS Software v2.3 and exported. Relative expression values were mean-centered, and heat maps were generated using the “Cluster” and “TreeView” software tools.

### 2.5. Bioinformatic Analyses

To avoid the limitation of a small sample size that may not provide a robust prediction, we utilized the TCGA database. The gene expression profile of GSE20916-RAW.tar was downloaded from Gene Expression Omnibus (GEO, http://www.ncbi.nlm.nih.gov/geo/). The GSE20916 dataset contained 145 samples, including 105 CRC tumor tissues and 40 normal colon epitheliums. The hierarchical clustering analysis was used to categorize the data into two groups that had similar expression patterns in CRC and normal colon epitheliums. We used a classical *t*-test to identify DEGs with a change ≥ twofold and defined *P* < 0.05 to be statistically significant. The Gene Ontology (GO) and Kyoto Encyclopedia of Genes and Genomes (KEGG) pathway enrichment analyses were performed to analyze the DEGs at the functional level. The DEGs were screened using Expression Console Affymetrix.

### 2.6. Validation of Target Gene Selection

Target genes were selected after analyzing the data from the TaqMan Array Plates and data mining with bioinformatics. Then, we validated the target genes using tumor tissues of 47 CRC patients. TaqMan quantitative PCR was used to determine the expression of the DEGs in the cancerous and adjacent noncancerous tissues (AXIN2 (ID. Hs01063168_m1), MMP-7 (ID. Hs01042796_m1), MYC (ID. Hs99999003_m1), WNT5A (ID. Hs00998537_m1), and GAPDH (ID. Hs99999905_m1), Thermo Fisher Scientific, USA). Specifically, amplified RNA from each sample was reverse-transcribed using SuperScript II reverse transcriptase and random primers (Invitrogen). cDNA from each reverse-transcribed sample was PCR amplified using TaqMan quantitative PCR. The quantification of the gene copy number, together with the human albumin gene, was performed with the 7500 Real-Time PCR System (Applied Biosystems, Courtaboeuf, France). The expression values for each gene were normalized with GAPDH.

### 2.7. Statistical Analyses

Statistical analyses were performed using SPSS (Version 10.0). Pearson's chi-squared analysis and Fisher's exact test were employed to compare the differences in categorical variables between patient groups. The relative gene expression levels were compared between various subgroups using the Kruskal–Wallis test (42 groups) and Mann–Whitney *U* test (two groups). DEGs were determined by Welch's *t*-test, and precise targets were selected from the DEGs using the prediction analysis for microarrays (PAM) software package. Significance was established when the statistical tests returned *P* values < 0.05. Software R (v3.1.1) was used to determine the significance in the TaqMan Array Plates, comparing genes expressed in CRC-HPV+ vs. CRC HPV tumor tissues (heat map).

## 3. Results

### 3.1. The Clinicopathological Characteristics of CRC Patients

To understand whether CRC patients associated with HPV would have unique features, we investigated their clinicopathological characteristics in this study; the parameters included age, gender, tumor location, histological grading, lymphatic metastasis, and clinical staging. The 47 patients included 4 cases of mucinous adenocarcinoma and 43 cases of nonmucinous adenocarcinoma. Males comprised a more significant proportion of CRC patients compared to females. The majority of tumor regions were located in the sigmoid colon and rectum (Table 1).

### 3.2. HPV Antigens in CRC Tissue and Adjacent Noncancerous Tissue

**Figure 1:**
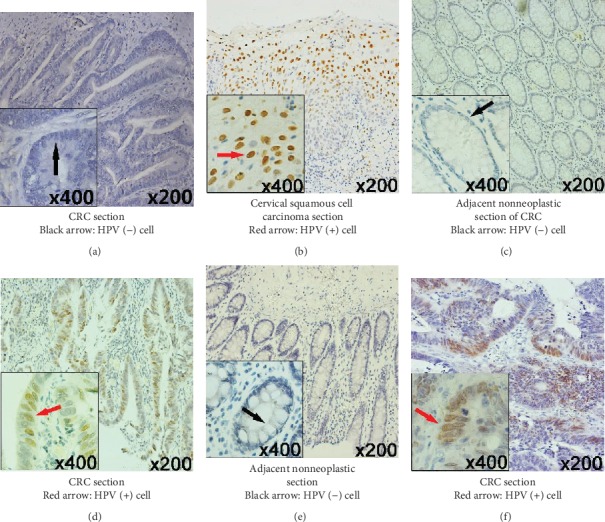
Distribution of HPV viral antigen in CRC tumor tissue and adjacent nonneoplastic tissue by immunohistochemistry staining. (a) HPV-negative expression in the neoplastic tissue of CRC patients with TNM stage 1. (b) HPV-positive squamous cell in cervical carcinoma was the positive control. (c, d) Patient 1 (TNM stage 3), with sigmoid colon cancer by which the pathological type is ulcerative moderately differentiated adenocarcinoma, and tumor cells infiltrate to the serous layer and adipose tissue. HPV staining is positive in tumor tissue (d) but negative in adjacent nonneoplastic tissue (c). (e, f) Patient 2 (TNM stage 3), with rectal cancer by which the pathological type is ulcerative moderately differentiated adenocarcinoma, and tumor cells infiltrate to the outer membrane; HPV staining is positive in tumor tissue (f) but negative in adjacent nonneoplastic tissue (e).

IHC staining showed that 15 (31.9%) cases were HPV positive, while 68.1% were HPV negative. HPV antigen was expressed in most cancerous tissues but not in adjacent noncancerous tissues in some CRC patients (Figure 1). At TNM stage I, all adjacent noncancerous tissues were viral negative, but at stage IV, half of the adjacent noncancerous tissues were viral positive. At stages II-III, HPV expression was significantly higher in cancerous tissue compared to adjacent noncancerous tissue. Our results indicated that cancerous tissues had higher levels of HPV expression than adjacent noncancerous tissue.

### 3.3. DEGs of HPV-Positive and HPV-Negative CRC Tissues

**Figure 2:**
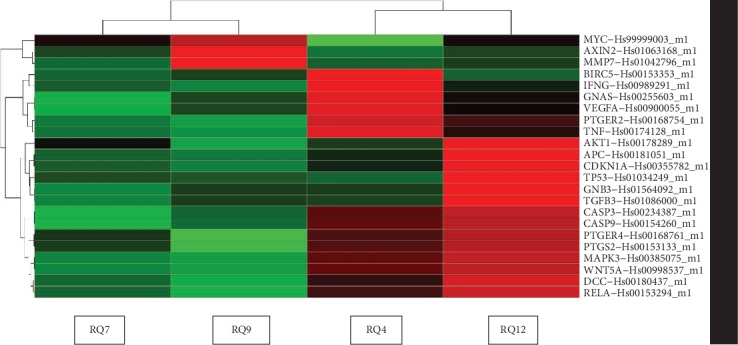
The heat map of 23 differentially expressed genes (DEGs) in HPV-positive and HPV-negative CRC tissues. RQ7 and RQ9 were tumor tissues with HPV negative. RQ4 and RQ12 were tumor tissues with HPV positive. The gene expression data were measured with microarray methods. Relative expression values (2-ΔCt) of DEGs from HPV-positive/negative sample were calculated and generated the heat map. DEG expression was shown with different colors. From green to red represent downregulated to upregulated genes.

We randomly selected four CRC patients, including two patients with HPV positive and two patients with HPV negative, analyzing ninety-two genes with the TaqMan Array Plates method. Cancerous and adjacent noncancerous tissues (paired samples) were used for the case-matched studies. Gene expression profiles in case-matched groups were analyzed using TaqMan Array Plates to screen DEGs (enumerated in Table 2). Gene expression levels are relative to GAPDH expression, where GAPDH expression level is equal to 1. Among the ninety-two genes, eleven were identified as twofold upregulated between HPV-positive and HPV-negative groups: *APC*, *CASP3*, *CDKN1A*, *IFNG*, *MAPK3*, *MAP2K4*, *NOS2*, *PTGS2*, *PTGER4*, *SMAD2*, and *TP53*. Twelve genes increased between onefold and twofold, namely, *ARRB1*, *CASP9*, *DCC*, *EGF*, *FZD1*, *IFNGR1*, *KRAS*, *JAK1*, *MAP2K2*, *MAP2K6*, *NFKB1*, and *TNF*. Sixteen genes increased between 0.5-fold and onefold, namely, *AKT1*, *GNA5*, *GNB3*, *IL-6R*, *KRAS*, *NFKB2*, *PRKACA*, *PTGS2*, *RELA*, *SMAD4*, *STAT1*, *TGF4*, *TGFB1*, *TGFBR1*, *TLR4*, and *WNT5A*. On the contrary, In the HPV-positive group, seventeen genes were downregulated between 0-fold and 0.5-fold, namely, *BAX*, *BCL2L1*, *CCND1*, *CCND2*, *CDH1*, *CTNNB1*, *DVL1*, *GRB2*, *LRP5*, *MLH1*, *MMP7*, *MMP9*, *MYC*, *PIK3R1*, *PIK3R2*, *SRC*, and *TCF3*. Five genes decreased between 0.5-fold and onefold, namely, *AXIN2*, *MAPK1*, *MSH2*, *PIK3CA*, and *LRP6*. The heat map of DEG expression (upregulated and downregulated genes) is shown in Figure 2. Our data suggested that the deregulation of the genes may play essential roles in HPV-associated CRC carcinogenesis. GO analysis results showed that DEGs were significantly enriched in biological processes including cell cycle, cell division, cell proliferation, immune response, intracellular signaling cascade, and defense response.

### 3.4. Bioinformatics Analyzed the DEGs between CRC Tumor Tissue and Nontumor Tissue

**Figure 3:**
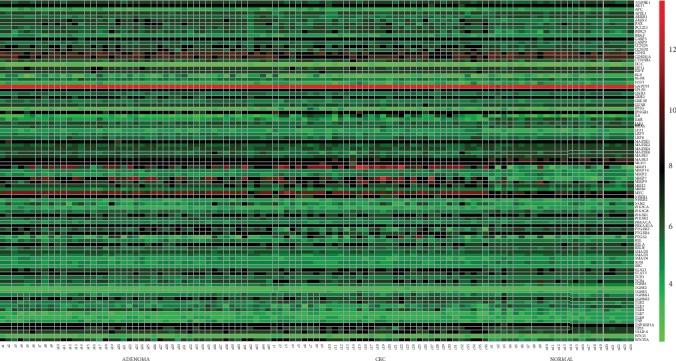


Additionally, we downloaded 145 raw CEL files from the NCBI Gene Expression Omnibus (GEO) database and normalized them with GeneChip RMA (GCRMA). Gene expression data were normalized for each tissue type by computing the Robust Multichip Average (RMA) directly from the Affymetrix. CEL were files obtained for tumors, adenoma, and healthy samples. A total of 3500 genes were identified, and the oncogenes associated with CRC were in the range. From these data, we found 16 oncogenes with a significantly different expression between the tumor and healthy tissues. Compared with healthy tissue, nine genes were upregulated between onefold and twofold in the CRC group: *MMP1*, *MMP7*, *MMP9*, *WNT5A*, *IL-6*, *PTGS2*, *MYC*, *BIRC5*, and *CCND1*. Seven genes upregulated between 0.5-fold and onefold: *AXIN2*, *VEGFA*, *TLR2*, *STAT1*, *LEF1*, *MEH2*, and *TLR4*. The downregulated genes (0.5-fold to 1.0-fold) include *IL-6R*, *TLR3*, *PIGER2*, *MAP2K6*, *ARRB1*, *PTGER4*, *CDKN1A*, *MAPK3*, and *TLR7*. The DEG expression heat map is shown in Figure 3. When adenoma is compared with the healthy tissue, we found that six genes increased between onefold and twofold: *MMP1*, *MMP7*, *WNT5A*, *MYC*, *AXIN2*, and *TLR4*, while six genes increased between 0.5-fold and onefold: *MSH2*, *TP53*, *NOS2*, *CCND2*, *BAX*, and *PTGER2*. The downregulated genes include *IL-6R*, *TLR3*, *ARRB1*, and *TLR7*.

### 3.5. Target Gene Validation

**Figure 4:**
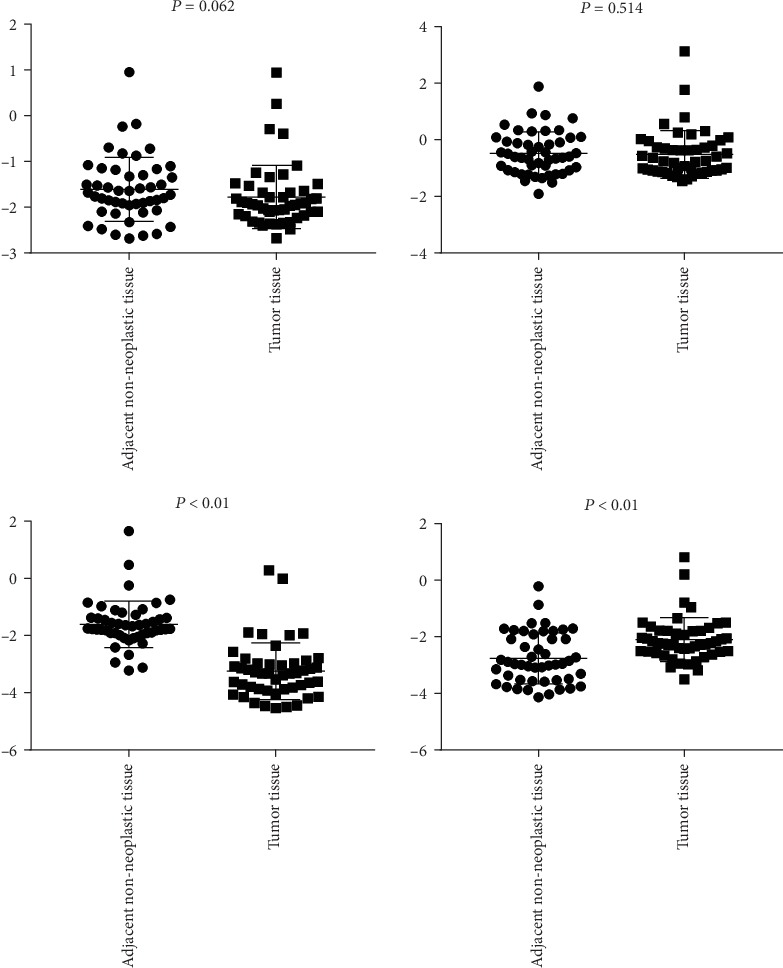
Expression of AXIN2, MYC, MMP7, and WNT-5A in CRC tissue and adjacent noncancerous tissue.

**Figure 5:**
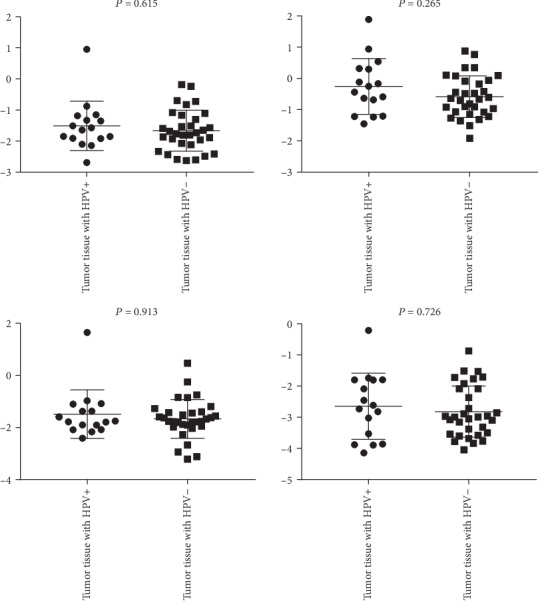
Different expression levels of AXIN2, MYC, MMP7, and WNT-5A between HPV-positive and HPV-negative tumor tissues.

To expand the sample size, the TCGA database was exploited to search the DEGs with significantly different expression in CRC and non-CRC tissues; combining with the DEGs from our microarray analysis, we found that *MMP7*, *WNT5A*, *MYC*, and *AXIN2* genes upregulated in tumor vs. healthy tissues, adenoma vs. healthy tissue in TCGA; these genes were overlapped between the TaqMan Array Plates data and TCGA data. To validate the results, we detected them in the 47 samples of CRC using the qRT-PCR assay. For all the four genes, the results were concordant with the TaqMan Array Plates data. Initially, a comparison was made between cancerous tissue and adjacent noncancerous tissue. The results showed that *MMP-7*, *MYC*, *WNT-5A*, and *AXIN2* were upregulated in cancerous tissue compared to adjacent noncancerous tissue, but only *MMP-7* and *WNT-5A* reached statistical significance (*P* < 0.05) (Figure 4). Then, we compared the same four genes between viral positive and negative groups. *MYC*, *WNT-5A*, and *AXIN2* were shown to be upregulated in HPV-positive CRC tissues when compared to HPV-negative tissues, while *MMP7* was shown to be downregulated in HPV-positive CRC tissues when compared to HPV negative tissues, but the difference did not reach the level of statistical significance (*P* > 0.05) (Figure 5).

### 3.6. DEGs' Principal Component Analysis

**Figure 6:**
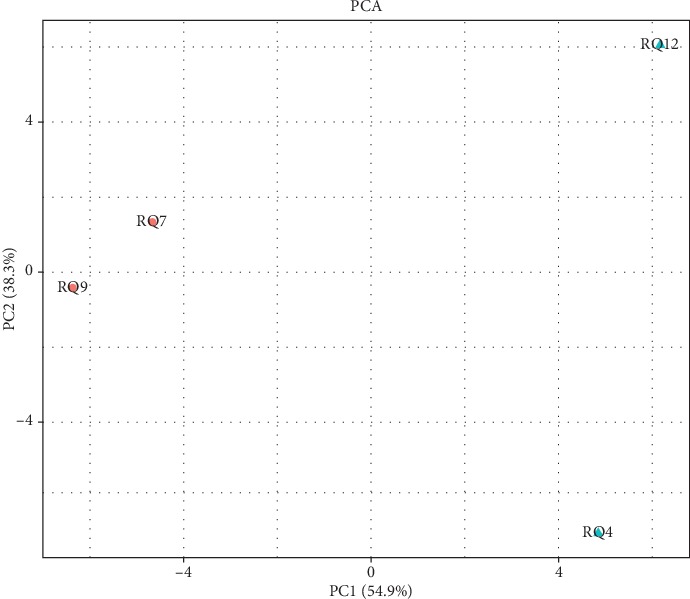
Principal component analysis (PCA) of gene expression data for HPV-positive and HPV-negative CRC tissues. RQ7 and RQ9 were tumor tissues with HPV negative. RQ4 and RQ12 were tumor tissues with HPV positive. 4 CRC samples (2 from viral-positive group and 2 from viral-negative group) were significantly separated into two areas.

**Figure 7:**
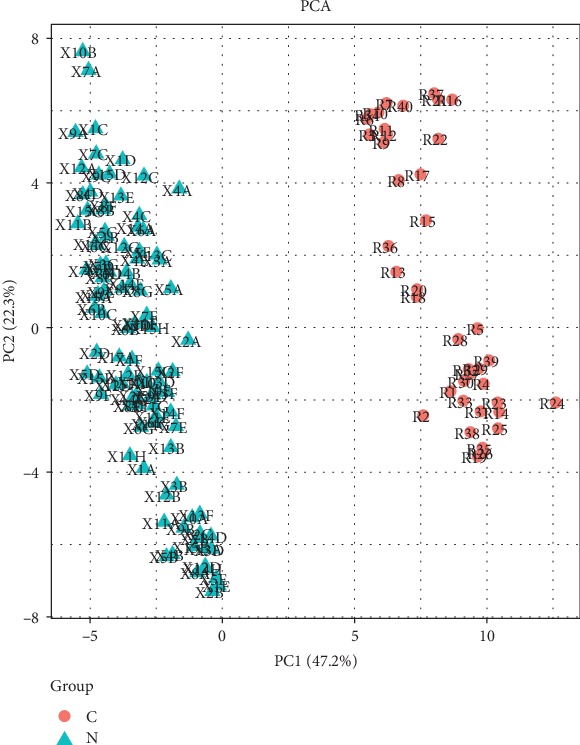
Two-way hierarchical clustering analysis of the data downloaded from TCGA. Group C for CRC tissues and Group N for normal tissues. The results showed that 92 genes were significantly separated into two areas: tumor tissue vs. normal tissue.

We then applied the hierarchical clustering analysis and principal component analysis (PCA) to categorize all the genes into different groups. Unsupervised two-dimensional hierarchical cluster analysis of relative gene expression data (2DCt method) reveals distinct clusters of cancer samples and healthy control samples. With PCA analyses, the 92-gene signature discriminated four samples into two significantly separated areas: viral-positive and viral-negative groups. Our results revealed that isolated areas considerably accurately classify the patterns of viral-positive and viral-negative CRC (Figure 6). The two-way hierarchical clustering was also used in the data from TCGA, and the results revealed significantly separated areas between CRC cancer tissue and healthy tissues (Figure 7). Our data show that multiple genes participate in the process of viral-related CRC.

## 4. Discussion

The human microbiota has implicated in the etiology of CRC; moreover, the microbiome has proven to be an active contributor to CRC [3]. FadA protein, a virulence factor expressed by *Fusobacterium nucleatum*, can signal epithelial cells via E-cadherin, a cell-surface molecule essential for CRC metastasis as well as a component of the WNT/*β*-catenin signaling pathway, the most commonly mutated pathway in CRC [34, 35]. Nowadays, extensive studies have revealed the close relationship between gut dysbacteriosis and CRC, but few have demonstrated the relationship between persistent viral infection in the gut and carcinogenesis of CRC. Recently, HPV antigens have been found in CRC tumor tissue; however, its pathogenesis remains to be elucidated. The molecular pathways that link HPV infection and CRC are still undetermined.

Comparative analysis of the DEGs reported by independent studies shows a relatively limited degree of overlap due to variations in population and technical methods [36, 37]. Therefore, it is still trying to delineate the underlying genetic events for individual CRC patients precisely. TaqMan Array Plates technology provides a highly effective way to analyze the expression changes of mRNA in CRC [38]. In this study, the DEGs detected by TaqMan Array Plates were related to colorectal carcinogenesis, cell cycle progression, invasion, antiapoptosis, cell adhesion and proliferation, and carbohydrate metabolism. Specifically, we found that 39 DEGs were upregulated, while 17 DEGs were downregulated when comparing HPV-positive CRC tissues with HPV-negative tissues. The DEGs in HPV-positive tissues were involved in signaling pathways including activators of Wnt/*β*-catenin, phosphoinositide 3-kinase (PI3K), mitogen-activated protein kinase (MAPK), mammalian target of rapamycin (mTOR) signaling, and inhibitors of TGF*β* and TP53 signaling, which were also the most frequent changes in CRC progression. The DEGs are also involved in cell invasion, adhesion, and tissue remodeling which may play an essential role in the carcinogenesis and progression of metastases in CRC [34, 39–41]. With PCA analyses, we found that genes expressed in viral-positive and viral-negative samples have located in distinctly different areas, which might accurately classify viral-positive- and viral-negative-related CRC DEGs. Our data is partially overlapping with TCGA data. TCGA data showed that DEG expression among CRC, adenoma, and healthy tissue was different. DEGs in CRC tumor tissue have also significantly separated areas with genes expressed in healthy tissue.

The Wnt/*β*-catenin signaling pathway controls cell proliferation and differentiation in the intestinal crypt microenvironment [42]. In the intestinal epithelium, terminally differentiated intestinal epithelial cells (IECs) were constitutively replaced by newly divided IECs from intestinal stem cells (ISCs) located in the crypts. This biological process was tightly controlled by Wnt signaling [43]. Deregulations of Wnt/*β*-catenin signaling were associated with CRC [44, 45]. The APC protein, encoded by the *APC* gene, plays a vital role in maintaining homeostasis of the gut epithelium and thus, when mutated, can impart a functional consequence capable of driving cancerous cell proliferation [46]. In this study, APC and WNT5A were upregulated in the HPV-positive group as compared to the HPV-negative group, indicating that HPV infection may activate the Wnt/*β*-catenin signaling pathway. Evidence from the previous study also supports that microbes play a role in dysregulating the Wnt/*β*-catenin signaling pathway as a means of tumor initiation [47]. AXIN proteins act as tumor suppressors that function through inhibition of Wnt signaling [48]. AXIN2 is a primary transcriptional target of *β*-catenin–dependent Wnt signaling, which acts as a scaffold factor in the *β*-catenin destruction complex. The two AXIN proteins have similarity in dysregulation of the Wnt pathway that believed to be an essential first step in the genesis of CRCs, while aberrant Wnt pathway activation is in roughly 90% of sporadic CRCs [34, 49].

In our study, we found that WNT5A was upregulated while AXIN2 was downregulated in the HPV-positive group as compared to the HPV-negative group. However, both AXIN2 and WNT5A were upregulated in the HPV-positive group when we verified the results with qPCR in additional samples. The data from TCGA showed that AXIN2 was upregulated in CRC tumor tissues. Our results revealed that differences in gene expression might be extended in individual patients with CRC; therefore, a large cohort is required to eliminate a distinctive difference in CRC oncogenes. The differences between microarray and q-PCR should be carefully examined to reveal the role of AXIN and Wnt signaling in HPV-associated CRC pathogenesis. Genes mutations in the Wnt/*β*-catenin signaling pathway include the c-MYC proto-oncogene (MYC) [50]. c-Myc is a target of APC signaling, which aberrantly activated in most colorectal cancers [51]. With the qPCR method, we found that MYC upregulated in all CRC cancerous tissues including HPV-positive cancerous tissues. MYC encodes a transcription factor that stimulates cellular proliferation and growth by controlling the expression of genes whose products regulate metabolism, ribosome biogenesis, and cell cycle progression [52].

Interferon (IFN) has a wide range of biological functions, including antiviral, antiproliferative, and immunomodulatory properties. IFN-induced cellular antiviral response is the primary defense mechanism against viral infections. Consequently, viral infection may disrupt the secretion of interferon, *IFNG*, *INFGR1*, and *TNF* genes upregulated in HPV-positive CRC tissues as compared to HPV-negative CRC tissues in this study. The upregulated *IFNG* and *INFGR1* may trigger the STAT (signal transducers and activators of transcription) proteins that mediate some biological functions and form part of the signaling cascades [53]. Therefore, the intimate relationship between viral infection, *IFN/TNF/IL-6R/STAT1*expression, and the associated signaling pathway is valuable in elucidating the mechanism of virus-associating CRC.

Striking experimental data have shown that gut microbes play a crucial role in vascularization of the intestinal mucosa and wound-healing processes [54]. MMPs and VEGFA are the most critical prometastatic and proangiogenic factors that inhibit cancer cell apoptosis, decrease cell adhesion, and induce angiogenesis, resulting in the promotion of the development and progression of CRC [55]. KRAS is a small GTPase, playing a significant role in the EGFR-MEKMAP kinase pathway. KRAS mutations are detectable relatively early in colon carcinogenesis, which is relevant to tumor progression and seems to be associated with distant metastases [56]. The PI3K/Akt signaling pathway affects diverse cell activities such as proliferation, differentiation, migration, and apoptosis, which is a crucial antiapoptosis pathway that regulates cell survival and death [57]. PI3K/Akt signaling also mediates inflammatory reactions by interacting with NF-*κ*B [58]. PTEN is a molecular switch that antagonizes the PI3K/Akt signaling pathway by dephosphorylating PIP3 to PIP2, which suppresses cell proliferation and promotes apoptosis. Downregulation of PTEN results in an enrichment of PIP3 and a high expression of Akt [59]. In this study, we also found that *MMPs*, *VEGFA*, *JAK1*, *EGF*, *KRAS*, *AKT1*, *MAPK3*, and *MAP2K4* were upregulated in HPV-positive CRC. The functions of these genes in virus-associated CRC need further investigation.

Currently, traditional clinical and pathological parameters are not always sufficient to discriminate high-risk from low-risk CRC, and validated molecular markers with prognostic value are not yet available [33]. A recent report demonstrated that analysis of the microbiome could serve as a screening biomarker for CRC [60]. High-risk HPVs are present in 53.84% of invasive colorectal cases, which is associated with an invasive and metastatic phenotype [61]. E6/E7 of HPV type 16 induces cellular transformation and migration in human healthy colorectal mesenchymal cells but not epithelial ones, accompanied by the upregulation of D-type cyclins and cyclin E as well as Id-1 in these cells [62]. Our results revealed that the HPV antigen was detected more strongly in tumor tissue than in adjacent nonneoplastic tissue. Moreover, all adjacent noncancerous tissues that were viral negative at TNM stage I were changed into half of the adjacent noncancerous tissues that were viral positive at stage IV. Our results indicated that HPV might be associated with the CRC stage. When viral proteins begin to accumulate, progeny viral genomes are replicated and used for secondary transcription. HPV integration sites, with a preference for sites of known genomic fragility, are distributed randomly over the whole genome in one study, and the majority of integrated HPV genomes appear to be actively transcribed [63]. In this pilot study, DEGs involved various signaling pathways. A larger cohort to identify genes from HPV-associated CRC is necessary to obtain comprehensive knowledge of the signaling pathways associated with CRC carcinogenesis, discover novel targets for cancer therapy, and develop new biological drugs [33].

The main limitation of this study is that no significance was observed between deregulated genes from HPV-positive vs. HPV-negative CRC cases of the cohort. We could not exclude the reason for the small sample size. However, there may be a more complicated mechanism involved. Further study with large sample size is needed to elucidate the HPV-associated CRC.

## 5. Conclusions

HPV-induced cervical cancer has been widely confirmed clinically, and the anti-HPV vaccine has made significant progress in the prevention and treatment of cervical cancer. At present, studies have found that HPV viral antigens also exist in colon cancer tissues. The relationship between HPV infection and CRC carcinogenesis is very worth studying. In this study, we demonstrated that tumor tissues had higher levels of HPV antigen than adjacent nonneoplastic tissue. Moreover, we found that 39 differentially expressed genes (DEGs) were upregulated, while 17 DEGs were downregulated when compared to HPV-positive CRC tissues with HPV-negative tissues. Combined with the database of the Cancer Genome Atlas (TCGA), we screen four DEGs and tested with the PCR method, and there was some overlap between our data and TCGA data. Our data revealed that HPV could take part in CRC carcinogenesis through gene mutation. The further genomic and proteomic investigation is necessary for obtaining a more comprehensive knowledge of signaling pathways associated with CRC carcinogenesis.

## Figures and Tables

**Table 1:** The clinicopathological characteristics of CRC patients.

	Total patients (*n*)
Cases of patients	47
Gender (male/female)	28/19
Median age (years) (range)	64 (38-82)
Location	
Right side of colon	1
Descending colon	1
Ascending colon	3
Transverse colon	6
Sigmoid colon	16
Rectum	19
Hepatic flexure	1
Clinical stage	
I	1
II	23
III	19
IV	4
Adenoma histological grade	
II	40
III	7
Mucinous/nonmucinous adenocarcinoma	4/43
HPV positive/HPV negative	15/32

**Table 2:** Gene expression profiles in case-matched groups.

Gene	TCGA				96-array	
	Normal tissue (FC)	Tumor tissue (FC)	ΔCT	*P*	HPV-(FC)	HPV+(FC)
ADRBK1	7.29	6.41	0.88	<0.001	0.89	1.13
AKT1	8.20	8.20	0.00	0.959	0.87	1.77
APC	5.60	4.30	1.30	<0.001	1.13	3.55
APPL1	9.19	6.66	2.53	<0.001	0.99	1.23
ARRB1	7.71	5.97	1.74	<0.001	1.62	3.15
AXIN2	7.11	6.31	0.80	<0.001	1.18	0.20
BAX	5.08	6.65	1.57	<0.001	1.12	1.11
BCL2L1	6.63	6.14	0.50	<0.001	0.80	0.55
BIRC5	7.70	6.35	1.35	<0.001	0.39	1.17
BRAF	7.45	4.58	2.87	<0.001	1.11	1.13
CASP3	10.04	8.16	1.88	<0.001	1.64	4.50
CASP9	7.17	5.61	1.56	<0.001	0.80	2.19
CCND1	8.66	7.13	1.53	<0.001	0.59	0.53
CCND2	7.75	6.64	1.11	<0.001	1.12	0.87
CDH1	9.20	9.19	0.02	0.862	1.68	1.57
CDKN1A	10.28	9.23	1.04	<0.001	1.12	3.35
CTNNB1	7.09	6.26	0.83	<0.001	0.89	0.87
DCC	3.84	2.86	0.97	<0.001	1.13	2.31
DVL1	8.34	6.84	1.50	<0.001	0.88	0.87
E2F4	8.22	7.09	1.13	<0.001	0.80	0.86
EGF	7.82	3.99	3.83	<0.001	1.76	3.42
EGFR	7.32	5.71	1.61	<0.001	2.18	2.21
FZD1	5.84	4.23	1.61	<0.001	1.73	3.04
GNAS	9.25	7.91	1.34	<0.001	0.80	1.60
GNB3	7.00	5.62	1.38	<0.001	0.82	1.76
GRB2	9.74	7.65	2.09	<0.001	1.63	1.62
GSK3B	7.68	5.44	2.24	<0.001	0.82	1.11
IFNG	5.71	3.51	2.19	<0.001	0.43	2.58
IFNGR1	9.23	7.86	1.37	<0.001	1.56	2.61
IL6R	6.57	5.06	1.50	<0.001	2.24	3.17
JAK1	7.10	6.28	0.82	<0.001	1.13	2.23
KRAS	7.99	6.13	1.86	<0.001	2.24	3.21
LEF1	5.25	4.43	0.82	<0.001	0.22	0.37
LRP5	5.58	5.37	0.21	<0.001	1.12	1.10
LRP6	8.06	5.88	2.17	<0.001	1.59	1.11
MAP2K1	10.33	8.26	2.07	<0.001	1.11	1.12
MAP2K2	6.74	6.26	0.48	<0.001	1.63	3.21
MAP2K4	8.47	6.61	1.86	<0.001	2.33	4.56
MAP2K6	7.57	6.07	1.50	<0.001	1.59	2.60
MAPK1	8.09	6.41	1.68	<0.001	2.31	1.72
MAPK3	9.55	8.51	1.04	<0.001	1.11	4.43
MLH1	10.75	8.29	2.46	<0.001	1.12	0.80
MMP14	6.72	5.24	1.48	<0.001	0.28	0.40
MMP2	5.50	5.12	0.39	<0.001	0.56	0.75
MMP7	7.73	7.85	0.12	0.766	0.13	0.03
MMP9	6.69	7.55	0.87	<0.001	0.81	0.66
MSH2	8.74	7.00	1.74	<0.001	1.61	1.12
MSH6	5.65	5.19	0.46	<0.001	0.80	0.85
MYC	10.92	9.92	0.99	<0.001	0.41	0.17
NFKB1	8.55	7.84	0.72	<0.001	1.11	2.18
NFKB2	4.66	4.45	0.21	<0.001	0.81	1.49
NOS2	6.56	5.51	1.05	<0.001	3.16	5.28
PIK3CA	6.56	4.32	2.23	<0.001	2.26	1.61
PIK3CB	7.50	5.64	1.85	<0.001	1.60	1.60
PIK3R1	8.27	7.53	0.73	<0.001	1.11	1.11
PIK3R2	7.61	6.07	1.54	<0.001	1.74	1.61
PRKACA	5.89	5.11	0.78	<0.001	1.61	2.24
PRKAR1A	9.03	7.94	1.09	<0.001	0.81	1.10
PTGER2	7.31	6.09	1.22	<0.001	0.87	6.43
PTGER4	8.90	7.71	1.18	<0.001	1.78	4.47
PTGS2	5.11	5.22	0.11	0.293	0.44	1.13
REL	6.54	5.31	1.23	<0.001	0.80	1.11
RELA	8.35	7.43	0.93	<0.001	0.54	1.11
RELB	6.23	5.79	0.43	<0.001	0.87	1.11
SMAD2	8.97	6.54	2.43	<0.001	2.27	4.47
SMAD3	6.46	5.47	0.99	<0.001	1.11	1.58
SMAD4	6.04	4.70	1.34	<0.001	1.58	2.23
SOS1	6.84	5.22	1.62	<0.001	1.60	1.73
SRC	5.53	4.72	0.81	<0.001	0.56	0.55
STAT1	8.36	7.26	1.10	<0.001	0.87	1.58
STAT3	7.96	7.35	0.61	<0.001	1.13	1.55
TCF3	7.04	5.59	1.45	<0.001	0.81	0.81
TCF4	7.49	6.86	0.63	<0.001	0.88	1.75
TGFB1	5.60	5.32	0.28	<0.001	0.56	1.11
TGFB2	4.42	3.24	1.18	<0.001	0.56	0.69
TGFB3	5.45	4.10	1.35	<0.001	0.41	0.86
TGFBR1	7.28	5.41	1.87	<0.001	1.11	1.64
TGFBR2	8.03	7.15	0.88	<0.001	1.61	1.75
TLR4	4.41	5.69	1.28	<0.001	1.13	1.75
TLR7	5.83	4.18	1.65	<0.001	1.73	2.26
TLR9	5.66	4.27	1.39	<0.001	0.81	1.11
TNF	4.33	4.58	0.25	<0.001	0.57	1.60
TNFRSF1A	9.46	8.36	1.10	<0.001	1.61	1.62
TP53	6.09	7.19	1.11	<0.001	0.55	2.61
VEGFA	7.27	7.10	0.17	<0.001	0.20	0.40
WNT5A	8.52	6.78	1.74	<0.001	0.28	1.13

Fold change of each sample calculated as follows: Normal/tumor tissue (FC) = 2^{−(CT(normal) − CT(internal reference))}^. HPV-/HPV+ group was the mean CT values of the cancer tissues minus noncancer tissue of the HPV negative or HPV positive. Due to the small number of samples, no intergroup comparison was made for the value of 96-array. The FC difference between HPV- and HPV+ was calculated based on >2 or <0.5 in CT.

## Data Availability

The data used to support the findings of this study are available from the corresponding author upon request.

## Abbreviations

CRC: Colorectal cancer
HPV: Human papillomavirus
IHC: Immunohistochemical
DEGs: Differentially expressed genes
TCGA: The Cancer Genome Atlas
RT-PCR: Reverse transcription-polymerase chain reaction.
